# UniteID– a proposal for training pediatric ID specialists supported by an adult ID program

**DOI:** 10.1007/s15010-025-02490-3

**Published:** 2025-03-03

**Authors:** Katrin Mehler, Andre Oberthuer, Gerd Faetkenheuer, Michael Weiss, Joerg Doetsch, Sarina K. Butzer, Norma Jung

**Affiliations:** 1 Department of Pediatrics, Pediatric Infectious Diseases, Faculty of Medicine, University of Cologne, Cologne, Germany; 2 Department I of Internal Medicine, Infectious Diseases, Medical Faculty, University of Cologne, Cologne, Germany; 3 Department of Pediatrics, Children´s Hospital Cologne, Cologne, Germany

**Keywords:** Infectious diseases, Training, Pediatrician, Pediatric infectious diseases

## Introduction

In light of rising antibiotic resistances and global pandemic challenges, migration and climate changes there is an increasing medical need for infectious diseases (ID) specialists to be involved in the diagnostic and treatment of infectious diseases in both adults and children. In a recent survey, US paediatric ID clinicians report that the volume and complexity of referrals increased over the recent years [1]. This is most likely due to the rising number of chronically ill, immunocompromised children and children with organ transplantation. Studies in adults have demonstrated that ID consultations improve appropriate antibiotic treatment and patient outcomes, increase compliance with existing guidelines and decrease costs [2]. Although, data on children are limited [3] similar effects are expected [4].

**Fig. 1:**
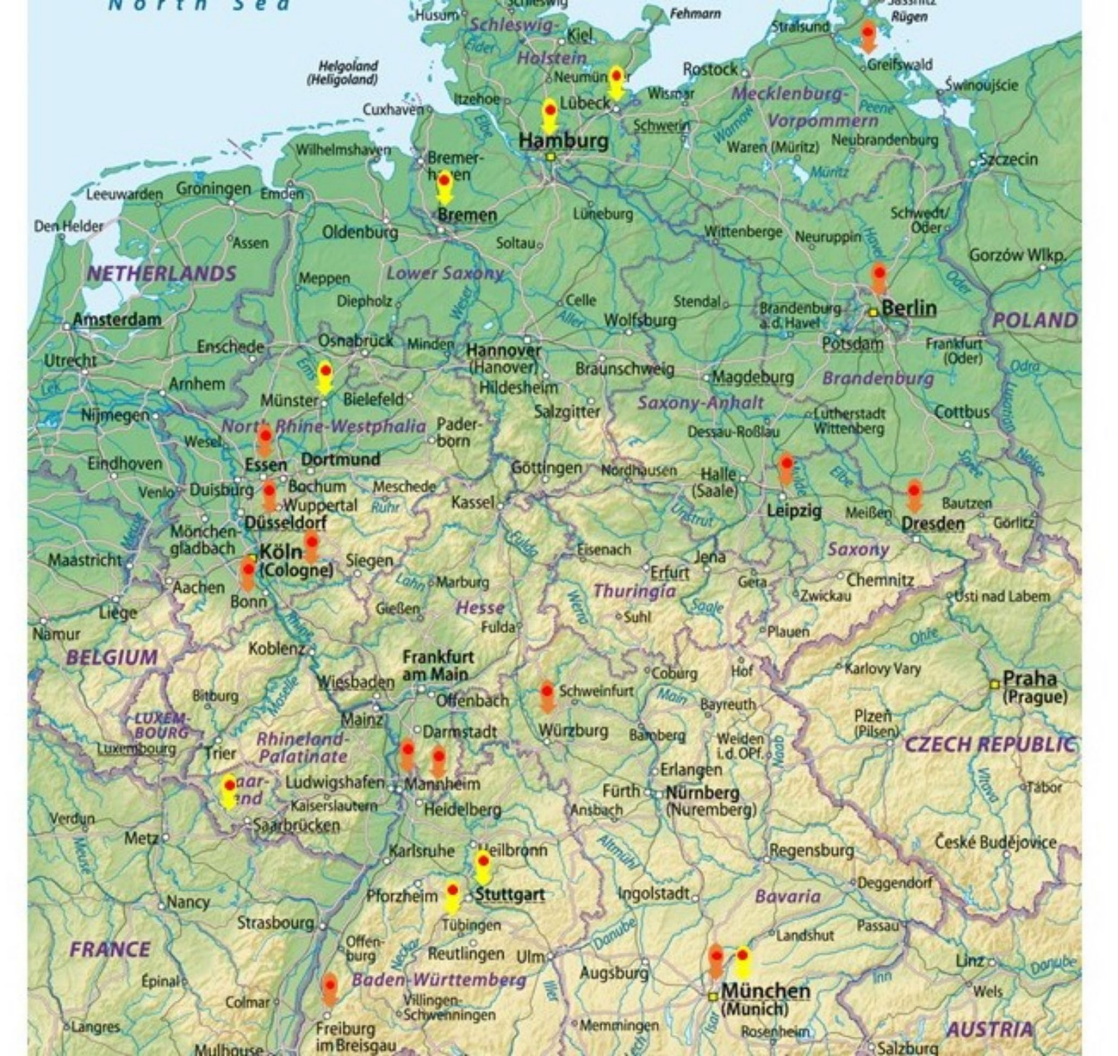
Map of the German pediatric centers that offer. -24 months pediatric ID training (orange markers) Leipzig and Dresden have a common center. -12 months general ID training (yellow markers)

Compared to the US, Canada, and many European countries, there is no nationwide pediatric infectious disease (PID) training accredited by the National Medical Association in Germany. However, the German Society of Pediatric Infectious Diseases (DGPI) offers a two-year PID program that is available in 12 out of approximately 300 hospitals with pediatric departments (Fig. 1, orange markers).

Moreover, state funding for ID training was recently terminated for pediatricians. These aspects make it increasingly difficult to train pediatricians in ID and antimicrobial stewardship (AMS) and emphasize the need for new strategies.

In line with that, no specialized pediatric ID training was available at the University Children´s Hospital of Cologne before 2015. It is one of the largest children’s hospitals in Germany with 130 beds and more than 5500 inpatient admissions per year. Specialized care is provided for all pediatric disease entities, including immunocompromised children from the department of pediatric oncology or with immunosuppression following solid organ transplantation. Before 2015, ID consultations in complex pediatric cases were performed on demand by adult ID clinicians trained in the ID section of the Department of Internal Medicine. Although basic ID principles are similar for children and adults, medical ID specialists may not be familiar with infections affecting newborns and young infants, respectively. Moreover, they may lack confidence in the correct choice, dosing, and side effects of antimicrobial drugs for children and may not be sufficiently skilled in infant physiology and diagnostic pitfalls. For example, in infants less than 12 months detection of *Clostridioides difficile* usually does not warrant treatment and most bone and joint infections need shorter intravenous and total duration of antibiotics compared to adults. Similarly, pediatricians are often less experienced in diseases that occur only infrequently in children, such as prosthetic infections, HIV or Hepatitis. Moreover, pediatricians are often less well informed about novel anti-infective drugs, which mostly are first applied in adult cohorts.

Considering this, we aimed to educate pediatric ID specialists with support of our adult ID department comprising both high clinical expertise and extensive basic and clinical research facilities. In this manuscript, we describe this approach highlighting both challenges and benefits for both adult and pediatric doctors. By providing an overview of this special concept we aim to encourage other centers to establish similar concepts to improve the expertise of both adult and pediatric ID clinicians, ultimately resulting in an improved care for patients.

## Practical implementation of pediatric ID training at the University of Cologne

The first step was to provide two skilled senior pediatricians working as consultants on the neonatal intensive care unit (NICU) with an ID training. In detail, an alternating rotation of two pediatric clinicians to the ID department was initiated. The adult ID division comprises an ID ward, an outpatient clinic and a special ID consultation service offering ID consultations (> 2500/ year) on demand for both, i.e. all departments at the University Hospital of Cologne, including pediatrics and some local community hospitals. Most consultations refer to highly complex infections in difficult treatment scenarios [5, 6]. In addition, weekly AMS ward rounds are conducted on all intensive care units at the University Clinic of Cologne. Furthermore, case discussions, lectures and expert sessions are held on a regular (usually weekly) basis. Overall, the ID consultation service offers training in treating complex infections under close supervision. To focus on infections in children we further established a close collaboration with a highly experienced pediatric ID specialist from a large community hospital in Cologne and arranged weekly meetings to discuss complex cases. The next steps involved applying to the German pediatric ID society (DGPI) for certification of a pediatric ID center based in the two pediatric hospitals mentioned above. This required the development of a 24-month specific training curriculum in accordance with the German version of the European guidelines for training pediatric ID specialists (https://dgpi.de/zusatzqualifikation-padiatrische-infektiologie). The program was adapted to match the specific conditions at our institution. The basic idea was to create a joint curriculum comprising both training with adult and pediatric ID specialists at the University of Cologne and the pediatric ID specialists at the large community hospital to fulfill the requirements of the European curriculum (https://www.espid.org/content.aspx?Group=education%26;Page=pid_training).

In 2022, the DGPI board approved our program. Our pediatric ID center was certified, and we started pediatric ID training in January 2023. Pediatric ID trainees receive training in pediatric as well as adult ID. For research, they are welcome to join the adult ID basic or clinical science labs and to transfer the methods and skills to a pediatric basic science lab. Finally, a pediatric ID outpatient clinic was established in May 2023. The steps and timeline are summarized in Table 1.

**Table 1:** Steps and timeline of pediatric ID implementation in Cologne

Implementation steps	Timeline
Training of two experienced pediatricians in ID	2015–2019
Board certification in infectious diseases	2020
Development of a joint curriculum	2021
*Mandatory*	
Clinical education	
• 6 months of ID consultation service • 6 months of ID training on the PICU and/or NICU • 3 months of ID training on a low/intermediate care pediatric ward treating children with infections	
External education	
• 1 month Institute for Medical Microbiology • 1 month Institute of Virology • 1 month Department for Infection Control and Hygiene • 2 weeks Public Health Department Cologne	
Research	
• 3–6 months of either clinical or basic science or translational ID project	
*Optional*	
• 3 months pediatric oncology (ward) • 3 months pediatric pulmonology (outpatient clinic) • 3 months adult ID (ward or outpatient clinic)	
Application for pediatric ID center certification	2022
Training of first pediatric ID fellows (24 months curriculum)	1/2022 to 12/2023
Opening of pediatric ID outpatient clinic	5/2023

PICU: Pediatric intensive care unit, NICU: Neonatal intensive care unit

*According to the guidelines issued by the German (DGPI) and the European (ESPID) societies of pediatric infectious diseases (https://dgpi.de/zusatzqualifikation-padiatrische-infektiologie)

The close collaboration of both adult and pediatric ID departments initiated more common projects. In detail, pediatricians were appointed to the University of Cologne AMS team and joined boards for special diseases including endocarditis and bone and joint infections. Moreover, pediatric outpatient parenteral anti-infective therapy (pOPAT) was implemented in children who needed intravenous treatment for more than two weeks and several manuscripts on infectious disease topics were drafted and published [7–11].

**Fig. 2:**
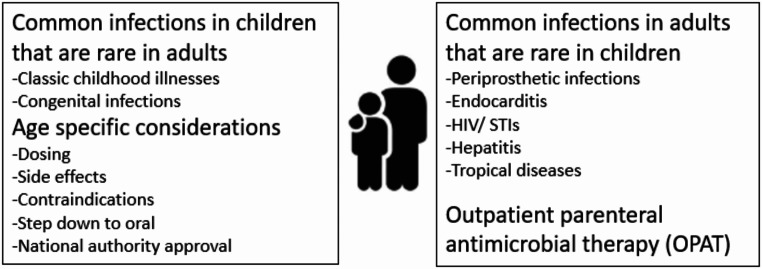
Specific expertise in pediatric and adult ID

By establishing and passing through this curriculum, important areas of expertise in pediatric and adult ID medicine was characterized (Fig. 2) that present training options for both, pediatric and adult ID specialists.

We observed that adult ID specialists’ confidence in giving recommendations was inversely correlated to children’s age with a high level of insecurity in extreme premature infants, in whom large differences in physiology, pharmacokinetics and clinical signs and symptoms were most evident. Similarly, adult ID specialists were often less experienced with clinical courses of classic childhood illnesses and recommendations for the duration of antibiotic treatment in infants < 3 years of age.

We conducted an online survey asking 10 adult ID specialists about their confidence in treating infections in children. The results are presented in Table 2. As expected, the confidence in diagnosis and treatment is slightly decreased with younger age.

**Table 2:** Online survey on confidence in pediatric ID

I feel confident in…	Children 3 ≤ years	Children > 3 years
ID treatment	3.0	3.4
Contraindications due to age	3.2	3.4
Dosing	2.9	3.4
Diagnostic limitations	2.8	3.4
Early step down	2.9	3.9
Duration of treatment	3.3	3.8
Classic infectious childhood illnesses	3.6	3.6

5 = extremely confident, 1 = not confident at all

In contrast, pediatricians have less experience in treating infections that are uncommon in children such as endocarditis or infections associated with prosthetic joints or implants. Moreover, the number of children presenting with HIV, tuberculosis or tropical diseases is low in European high resource countries. Consequently, knowledge exchange in diagnosis and treatment between pediatric and adult ID physicians is helpful. New antimicrobial substances for the treatment of complex infections take a long time to find their way into pediatric care although most have been used in adults for years.

In line with these observations, the importance of specialized pediatric ID clinicians is further emphasized by an analysis of all pediatric ID consultations that were done in the University of Cologne Children’s Hospital from October 2022 to October 2023. As shown in Table 3 344ID consultations were performed in 267 pediatric patients. Of these, almost one in two was in children below three years of age, thereby demonstrating that a major part of pediatric ID consultations occurs in the cohort of young children in which adult ID specialists feel least confident. Main indications for consultations < 3 years were late onset sepsis, enterovirus infections, urinary tract infections, fever of unknown origin, respiratory syncytial virus infections, cellulitis and lymph adenitis. For children > 3 years most consultation requests referred to bone and joint infection, appendicitis, invasive Streptococcus A infections, pneumonia and Mycoplasma infections. For all ages the most common consultations concerned urinary tract infections in chronic kidney disease, infections in immunocompromised patients, postoperative wound infection, severe acute respiratory infections in neurologically impaired children or young infants and Influenza.

**Table 3:** Number (percentage) of patients with pediatric ID consultations per age group in 12 months

Age group	*n* = 267
Children ≤ 3 years	130 (49%)
Newborn (1–30 days)	23 (9%)
Infant (1–12 months)	56 (21%)
Toddler (1–3 years)	51 (19%)
Children > 3 years	137 (51%)
Preschooler (4–6 years)	41 (15%)
Middle childhood (7–12 years)	39 (15%)
Teenager (12–18 years)	57 (21%)

## Discussion

With the support of adult ID specialists from a large ID department in Germany, the University of Cologne Children’s Hospital established a certified paediatric ID centre. As a result of this process both paediatric and adult ID specialists grew together to mutually learn about and contribute to ID treatment. Paediatricians were appointed to the hospital’s AMS team, offer joint ID education and started common basic and clinical research programs with adult ID clinicians. We formed a close collaboration between adult and paediatric ID for the profit of both sides and the patients. *A* combined training allows for gaining experience with a broader range of diseases. Moreover, new diagnostic tools and therapeutic options are often implemented first in adults. Of note, an age-based cut-off often does not reflect the patient´s physiology. The physiology of a teenager resembles that of an adult. However, an adult with a complex congenital condition may have the physiology of a child.

Our vision is to further expand and tighten our collaboration by forming new interdisciplinary boards addressing emerging issues, such as tuberculosis, which is on the rise due to the high numbers of refugees fleeing from Ukraine. We plan to merge our expertise to provide combined care to HIV infected mothers and their newborn infants addressing controversial topics such as breastfeeding. Regular meetings offer not only the opportunity to discuss unsolved or complex cases but work as a platform to discuss hot topics affecting both children and adults, such as the increasing number of Parvo B 19 infections or new vaccines for RSV. Furthermore, telemedicine will certainly play a major role in the future. In our centre no telemedical infrastructure has been implemented so far. However, a possible limitation of telemedicine is that the communication of clinical findings occurs through a third party. Tus, a thorough clinical examination by trained ID specialist, that may be essential in solving complex cases, is not possible.

To the best of our knowledge, the concept of a combined ID training program has not yet been published in Germany, despite its implementation in several tertiary care centres. In contrast, ID fellowships in the US, which are designed for both, adult and pediatric clinicians, are more widely recognized and actively promoted. (https://medpeds.org/fellowship-guide/infectious-disease). Moreover, it was advocated that adult and pediatric ID clinicians should combine forces in an ID “special interest group” [12].

Sufficient funding of ID training is a major issue in many countries that fortunately has gained political attention during the Covid-19 pandemic. In Germany, due to the initiative of the German ID society (DGI), the need for qualified ID specialists was met by a recent introduction of an extended training for internal medicine clinicians, which is funded to a major part by the German authorities. Unfortunately, within this framework, no pediatric ID specialization was introduced, and the funding program is not available. Currently, only about five clinicians per year are trained as pediatric ID specialists in Germany. This underscores the urgent need to expand both personnel and training capacities, including the implementation of a nationwide subspecialty training program in pediatric ID—an approach already implemented in many other countries.

. Lack of funding for physician-scientist careers in pediatric ID is a worldwide problem.

In Germany, this issue results in a shortage of available positions compared to the US where low salaries play a major role [12]. Consequently, almost one of two open fellowship posts in the US did not fill [13]. Alternative strategies, such as a common ID training may help to fill the gap.

## Conclusion

Diagnosing and treating infectious diseases comprises similar principles and challenges for both adult and children ID specialists. There are, however, substantial differences, which create opportunities for mutual learning. ID training programs in pediatrics need to be promoted politically and structurally, to foster collaboration. We are convinced that a close alliance between ID specialists improves the quality of patient care.

## Data Availability

No datasets were generated or analysed during the current study.
